# Death of Hon. N. J. Hammond

**Published:** 1899-05

**Authors:** 


IT is our sad duty to chronicle the death of N. J. Hammond, LL.D., of Atlanta, and in so doing we wish to add a few words in praise of his eminent qualities. For a number of years Col. Hammond was the President of the Board of Trustees of the old Atlanta Medical College, and when this was consolidated with the Southern Medical and made the present Atlanta College of

Physicians and Surgeons, by unanimous consent he was elected to the same position on the new Board. Colonel Hammond's address before the graduating class, the first to receive diplomas from the new consolidated institution, was delivered on the evening of April 3d. It is with sorrowful pleasure that we publish this address in the present issue. Sorrowful that we shall never again hear the silver tongue of this gifted man; a pleasure because we can present to the readers of THE JOURNAL-RECORD an address which will ever remain a classic. This was his last appearance in public, for two days later Colonel Hammond was taken sick, and this proved to be his final illness. He was at the time of his death President also of the Board of Trustees of the State University at Athens. Colonel Hammond served the Atlanta district in Congress several years ago, and was then recognized as one of the ablest representatives from the South. He was in the truest sense a cultured and intellectual man, and added to these was his well-marked Christian piety. In his death Georgia has lost one of her most gifted sons. His was a life that left its imprint behind him, and though his physical body will be no more, his works will ever remain a lasting monument to the true greatness of the man.

